# Cytotoxicity and Nitric Oxide Production Inhibitory Activities of Compounds Isolated from the Plant Pathogenic Fungus *Curvularia* sp.

**DOI:** 10.3390/jof7060408

**Published:** 2021-05-22

**Authors:** Virayu Suthiphasilp, Achara Raksat, Tharakorn Maneerat, Sarinya Hadsadee, Siriporn Jungsuttiwong, Stephen G. Pyne, Putarak Chomnunti, Wuttichai Jaidee, Rawiwan Charoensup, Surat Laphookhieo

**Affiliations:** 1 Center of Chemical Innovation for Sustainability, School of Science, Mae Fah Luang University, Chiang Rai 57100, Thailand; virayu.suthiphasilp@gmail.com (V.S.); achara6299@gmail.com (A.R.); wisanu.man@mfu.ac.th (T.M.); 2 Medicinal Plants Innovation Center of Mae Fah Luang University, Chiang Rai 57100, Thailand; wuttichai.jai@mfu.ac.th; 3 Center for Organic Electronic and Alternative Energy, Department of Chemistry and Center of Excellence for Innovation in Chemistry, Faculty of Science, Ubon Ratchathani University, Ubon Ratchathani 34190, Thailand; subaka29@gmail.com (S.H.); siriporn.j@ubu.ac.th (S.J.); 4 School of Chemistry and Molecular Bioscience, University of Wollongong, Wollongong, NSW 2522, Australia; spyne@uow.edu.au; 5 Center of Excellence in Fungal Research, School of Science, Mae Fah Luang University, Chiang Rai 57100, Thailand; putarak.cho@mfu.ac.th; 6 School of Integrative Medicine, Mae Fah Luang University, Chiang Rai 57100, Thailand

**Keywords:** *Curvularia* sp., *Dactyloctenium aegyptium*, curvulariahawadride, cytotoxicity, nitric oxide production inhibitory activity

## Abstract

Chemical investigation of the mycelia of the pathogenic fungus *Curvularia* sp. which was isolated from a leaf of *Dactyloctenium aegyptium* (crowfoot grass), resulted in the isolation of a new compound, curvulariahawadride (5), along with five known compounds (1–4, and 6). Their structures were determined on the basis of spectroscopic data, including 1D and 2D NMR and HRESIMS. The absolute configuration of 5 was established from experimental and calculated electronic circular dichroism (ECD). Compounds 1, 3, and 5 showed nitric oxide (NO) production inhibitory activity with IC_50_ values of 53.7, 32.8, and 12.8 µM, respectively. Compounds 2 and 4 showed significant cytotoxicity against lung cancer A549, colorectal cancer SW480, and leukemic K562 cells with an IC_50_ ranging value of 11.73 to 17.59 µM.

## 1. Introduction

Endophytic fungi are well-known as promising sources of structurally diverse and novel biologically active compounds [1,2,3]. Endophytic fungi have become one of the most important sources for drug discovery from nature because of the short period to scale up and the diversity of production of secondary metabolites [4,5]. *Curvularia* species are phytopathogenic fungi that are commonly isolated from soil or infected grasses and food plants, such as maize (*Zea mays*), oil palm (*Elaeis guineensis*), or rice (*Oryza sativa*) [6,7,8]. A major cause of leaf blight and leaf spot is infection by *Bipolaris* and *Curvularia* fungi and is considered one commonly associated with oil palm and corn leaf spots in Thailand [7,9]. In addition, several species of *Curvularia* have been reported for opportunistic infections in humans [10,11,12]. Previously, *C. lunata* was believed to be the most frequently reported human pathogenic species, while *C. hawaiiensis* (previously *Bipolaris hawaiiensis*) was reported to infect the ear of a patient post-trauma [12,13]. Previously, cochlioquinones, polyketides, terpenoids, alkaloids, quinones, and peptides have been isolated from the genus of *Curvularia* [14]. However, differences in fungal collections and fermentation conditions have yielded different compounds. Various types of compounds isolated from *Curvularia* species have shown impressive biological activities, including phytotoxic [15], antimicrobial [16,17,18,19,20], and cytotoxic [17,21,22] activities. Herein, we describe the isolation and structure elucidation of a new compound (**5**) along with five known compounds (**1**–**4**, and **6**) from the ethyl acetate extract of mycelia from laboratory cultures of *Curvularia* sp. which was isolated from a leaf of *D. aegyptium*. In addition, the in vitro nitric oxide (NO) production inhibitory and cytotoxicities of the isolated compounds against three cancer cell lines, including lung cancer A549, colorectal cancer SW480, and leukemic K562 cells, and mammalian cells (RAW 264.7) are also reported.

## 2. Materials and Methods

### 2.1. General Experimental Procedures

The UV spectra were recorded with a Varian Cary 5000 UV-Vis-NIR spectrophotometer. IR spectra were recorded on a Perkin Elmer FTS FT-IR spectrometer. The optical rotations were measured with a Bellingham and Stanley APD440 polarimeter. Electronic circular dichroism spectra were recorded on a JASCO J-815 spectrometer. The NMR spectra were recorded on a 400 MHz Bruker FT-NMR Ultra Shield. ESI-QIT-MS spectra were measured on a Bruker-Hewlett-Packard 1100 Esquire-LC system mass spectrometer. Quick column chromatography (QCC) and column chromatography (CC) were performed on silica gel C60 (0–20 µm, SiliCycle^®^ Inc., Québec, QC, Canada) and silica gel G60 (60–200 µm, SiliCycle^®^ Inc., Québec, QC, Canada), respectively. Sephadex LH-20 (25–100 µm, Merck, Kenilworth, NJ, USA), where indicated, was also used for CC. Precoated TLC plates of silica gel (60F254, Merck, Kenilworth, NJ, USA) were used for analytical purposes.

### 2.2. Fungal Material and Identification

A leaf spot symptom of crowfoot grass was collected from Hat Yai, Songkhla Province, in July 2012. Fresh specimens were incubated for 1–2 days in a moist chamber to induce sporulation. Fungi were isolated by a modified single spore suspension method [23]. Conidia were taken from the fungal sporulation from leaf spot samples and placed in sterilized water for spore suspension. The conidia were then transferred to water agar media and left overnight to germinate, and germinated conidia were individually transferred to PDA. The pure cultures of *Curvularia* sp. (MFLCC12-0192) were deposited in MFLUCC for future study. Genomic DNA was extracted from fungal mycelium grown on PDA media using the Biospin Fungus Genomic DNA Extraction Kit (BioFlux^®^, Hangzhou, China), following the instructions of the manufacturer. The DNA amplification was performed by polymerase chain reaction (PCR). Primers ITS1 and ITS4 (Glass and Donaldson, 1995) were used to amplify the 5.8S and ITS regions. The quality of PCR amplification was confirmed on 1% agarose gel electrophoresis stained with ethidium bromide. The amplified PCR fragments were sent to the commercial sequencing provider (Shanghai Sangon Biological Engineering Technology & Services Co., Shanghai, China).

### 2.3. Fermentation, Extraction, and Isolation

Fungal isolation *Curvularia* sp. (MFLCC12-0192) was grown on potato dextrose agar (PDA) (Millipore, Merck, Kenilworth, NJ, USA) at 25 °C for 5 days. Five pieces (0.5 × 0.5 cm^2^) of mycelial agar plugs were inoculated into Erlenmeyer flasks (2 L × 45), each containing 400 mL of PDB media which were allowed to stand at room temperature for 4 weeks. The culture was filtered to obtain the filtrate and mycelia. Wet mycelia were extracted twice with 500 mL of MeOH. After the concentration of the MeOH solution to 100 mL, H_2_O (100 mL) was added, and the mixture was extracted three times with EtOAc (200 mL each). The ethyl acetate layer was concentrated under reduced pressure to obtain a brown gum (2.4 g). The crude extract was subjected to quick column chromatography (QCC, 10 × 20 cm) over silica gel (0–20 µm), eluting with a gradient of EtOAc-hexanes (0:5, 0.5:4.5, 1:4, 1.5:3.5, 2:3, 2.5:2.5, 3:1.5, 3.5:1.5, 4:1, 4.5:0.5, 0.5:4.5, and 5:0, *v/v*, 250 mL each) to afford three combined fractions (F1–F3). Fraction F2 (375.8 mg) was further purified by Sephadex LH-20 CC (25–100 µm, 13 × 50 cm) using 100% MeOH (2000 mL) and then follow by repeated silica gel CC (60–200 µm, 12 × 50 cm) using acetone/hexanes (2:8, *v/v*, 1500 mL) to afford compounds **1** (3.7 mg), **2** (14.5 mg), **3** (2.3 mg), **4** (15.6 mg), and **6** (4.8 mg). Compound **5** (2.1 mg) was obtained from fraction F3 (264.9 mg) by Sephadex LH-20 CC (25–100 µm, 13 × 30 cm) using 100% MeOH (2000 mL) and follow by silica gel CC (25–100 µm, 12 × 50 cm) using MeOH/CH_2_Cl_2_ (0.6:9.4, *v/v*, 1000 mL).

### 2.4. Bioassays

#### 2.4.1. Nitric Oxide (NO) Production Inhibitory Assay

This assay was performed as previously described [24]. RAW 264.7 cells were seeded at 4 × 10^4^ cells/well in 96-well plates and incubated at 37 °C and 5% CO_2_ overnight. Cells were incubated with 1 µg/mL LPS for 1 h and treated with various concentrations of tea extract, including 3.125, 6.25, 12.5, 25, 50, and 100 µg/mL, for 24 h. After 24 h, 100 µL of Griess reagent was added to the samples for 10 min. Nitric oxide was measured at 570 nm with a Biochrom EZ Read 400 ELISA microplate reader (Biochrom Ltd., Cambridge, UK). Additionally, the data are presented as the IC_50_, which was calculated with GraphPad Prism 6.0 software. Indomethacin was used as a positive control with an IC_50_ value of 73.4 μM.

#### 2.4.2. Cytotoxicity Assay in Mammalian Cells (RAW 264.7 Cells)

This assay was performed as previously described [25]. Cell viability was measured by the MTT assay. RAW 264.7 cells were seeded at 4 × 10^4^ cells/well in 96-well plates and incubated at 37 °C and 5% CO_2_ overnight. Cells were treated with different concentrations of tea extracts, including 3.125, 6.25, 12.5, 25, 50, and 100 µg/mL, for 24 h. After 24 h, cells were washed with PBS and incubated with 0.5 mM MTT reagent for 4 h. The detection of formazan at 570 nm was performed with a Biochrom EZ Read 400 ELISA microplate reader (Biochrom Ltd., Cambridge, UK). The data were calculated as IC_50_ values with GraphPad Prism 6.0 software.

#### 2.4.3. Cytotoxicity Assay against Lung Cancer A549, Colorectal Cancer SW480, and Leukemic K562 Cells

These assays were performed as previously described [25]. Lung cancer A549 and colorectal cancer SW480 cells were maintained with Dulbecco’s modified Eagle’s medium (DMEM) containing 10% fetal bovine serum (FBS) and 1% penicillin/streptomycin. Leukemic K562 cells were cultured in RPMI-1640 supplemented with 10% fetal bovine serum (FBS) and 1% penicillin/streptomycin. All cells were cultured in 96-well plates at 37 °C in 5% CO_2_, followed by treatment with the sample for 24 h. After the incubation period, 0.5 mg/mL MTT was added to the cells and left for 4 h. The formazan was dissolved in DMSO and measured at 570 nm using Biochrom EZ Read 400 ELISA microplate reader (Biochrom Ltd., Cambridge, UK).

### 2.5. Computational Methods

The electronic circular dichroism (ECD) calculations of compound **5** were carried out by using TD-DFT at the CAM-B3LYP functional with 6-311++G(d,p) basis. All structures were optimized by the DFT method at the B3LYP/6-31G (d,p) level of theory. Geometry optimization and TD-DFT computations were both performed with Continuum Model (PCM) solvation model with methanol. The rotary strengths of 100 excited states were calculated. All calculations were performed using Gaussian 09 program package [26]. Gaussian band shape with a bandwidth of 0.25 eV was used to simulate ECD spectra. The ECD curves were generated by the software SpecDis 1.64 (University of Wurzburg, Wurzburg, Germany).

## 3. Results and Discussion

### 3.1. Isolated Compounds from Curvularia sp.

**Figure 1:**
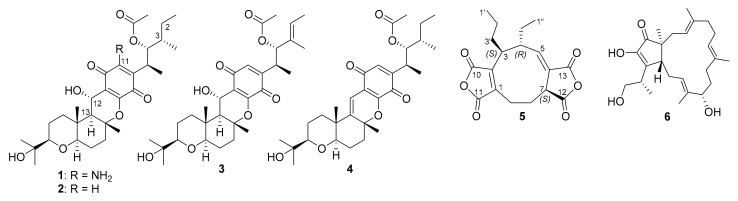
Compounds isolated from *Curvularia* sp.

The mycelia of *Curvularia* sp. were cultured on potato dextrose agar, and the mycelia were harvested, extracted, and subjected to repeated column chromatography to afford six compounds (Figure 1).

### 3.2. Structural Characterization of a New Compound

Curvulariahawadride (**5**), [α]_D_^25^: +65 (c 0.1, MeOH), was isolated as a colorless viscous oil and its molecular formula was assigned as C_18_H_20_O_6_ based on the ^13^C NMR spectroscopic data and HRESIMS, which showed an ion peak at *m/z* 333.1339 [M + H]^+^ (calcd 333.1338). Its UV spectrum showed the characteristic absorption of a maleic anhydride chromophores at λ_max_ 252 nm [27,28]. The IR spectrum indicated the stretching bands of hydroxy (3298 cm^−1^), anhydride carbonyl (1838 and 1763 cm^−1^), and carboxylic acid carbonyl (1713 cm^−1^) groups [27]. The ^13^C NMR (Table 1) and DEPT spectroscopic displayed 18 carbon resonances which were assigned to two methyls (δ_C_ 14.0 and 12.3), five methylenes (δ_C_ 34.9, 25.9, 24.3, 24.4, and 21.0), four carbonyls (δ_C_ 177.6, 169.8, 165.9, and 165.7), three sp^3^ methines (δ_C_ 46.3, 44.9, and 40.4), and four sp^2^ carbons (δ_C_ 148.3, 146.9, 142.7, and 131.2). The 1D and 2D NMR data (Table 1) identified the presence of an ethyl group [δ_H_/δ_C_ 1.98 (1H, ddd, *J* = 13.2, 7.2, 2.5 Hz, H-2′′a) and 1.38 (1H, m, H-2′′b)/25.9 and 0.93 (3H, t, *J* = 7.2 Hz, H-1′′)/12.4], a propyl group [δ_H_/δ_C_ 0.92 (3H, t, *J* = 7.6 Hz, H-1′)/14.0, 1.25 (2H, dd, *J* = 15.2, 7.6 Hz, H-2′)/21.0, and 1.89 (1H, m, H-3′a) and 1.53 (1H, m, H-3′b)/34.9], three methine protons [δ_H_/δ_C_ 2.82 (1H, td, *J* = 10.5, 2.7 Hz, H-3)/40.4, 2.68 (1H, m, H-4)/46.3, and 3.49 (1H, d, *J* = 12.0 Hz, H-7)/44.8], two pairs of diastereotopic methylene protons [δ_H_/δ_C_ 2.93 (1H, dd, *J* = 13.0, 7.3 Hz, H-9a) and 2.46 (1H, t, *J* = 13.0 Hz, H-9b)/24.4 and 2.69 (1H, m, H-8a) and 1.76 (1H, dd, *J* = 13.0, 12.6 Hz, H-8b)/24.7], and an olefinic proton [δ_H_/δ_C_ 6.46 (1H, d, *J* = 11.7 Hz, H-5)/148.3]. Analysis of the 2D NMR spectra suggested that curvulariahawadride (**5**) had a polyketide nonadride skeleton.

**Figure 2:**
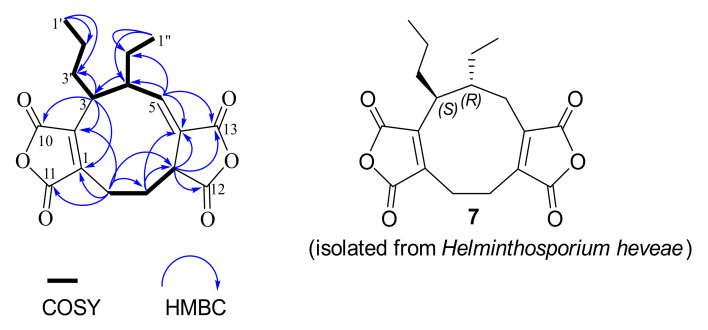
COSY (^1^H—^1^H) and selected HMBC (^1^H→^13^C) correlations of **5.**

**Figure 3:**
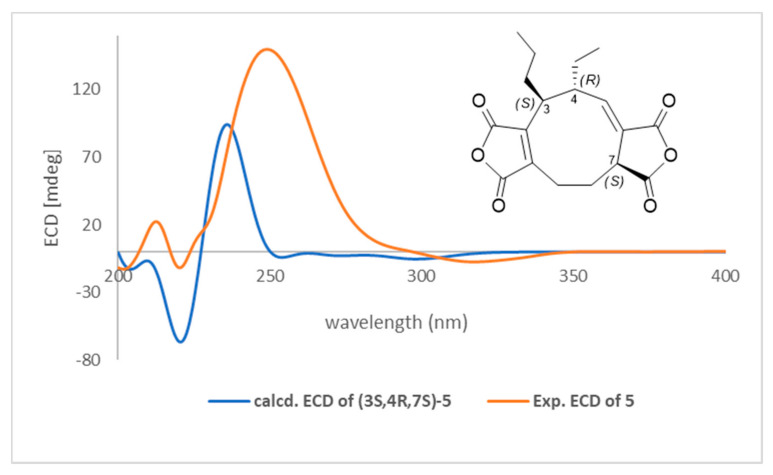
ECD spectra of compound **5**. Experimental and TDDFT calculated ECD spectra of compound **5** at CAM-B3LYP/6-311++G(d,p) level in methanol (PCM).

Key HMBC correlations (Figure 2) of H-3 with C-1, C-5, C-10, C-2′, and C-2′′ and H-9 with C-1, C-2, and C-11 suggested a five-membered anhydride nucleus on C-1 and C-2. COSY cross-peaks (Figure 2) between H-3/H-4, H-3/H-3′, H-4/H-5, and H-4/H-2′′ confirmed the attachment of the ethyl and propyl substituents to C-4 and C-3, respectively. From the above information, it was apparent that compound **5** is a nonadride derivative, which has a unique nine-membered carbocyclic ring, previously found in heveadride (**7**) [27,28]. The correlations between H-3/H-2b′′, H-4/H-1’, H-4/H-2’, H-5/H-3, H-5/H-1′′, H-5/H-2b′′, and H-7/H-9b in the NOESY experiment, suggested that H-3 and H-7 were located in the same side (α-orientation) but opposite to that of H-4 (β-orientation) (Figure S4). Moreover, the magnitude of the coupling constant (*J* = 10.5 Hz) of H-3 (δ_H_ 2.82) and H-4 (δ_H_ 2.68) suggested that these protons were in trans-orientation [27,28]. Finally, the absolute configuration of **5** was determined from the experimental and calculated electronic circular dichroism (ECD), as shown in Figure 3. Six putative structures of compound **5** (Figure S18) were optimized using B3LYP/6-31G(d,p) level in methanol (PCM) and the ECD spectrum for (3*S*,4*R*,7*S*)-**5**, (3*R*,4*R*,7*S*)-**5**, (3*S*,4*S*,7*S*)-**5**, (3*S*,4*S*,7*R*)-**5**, (3*R*,4*S*,7*S*)-**5**, and (3*R*,4*S*,7*R*)-**5** were calculated at CAM-B3LYP/6-311++G(d,p) level in methanol (PCM) (Figure S19). Of these, the experimental ECD spectrum of **5** was similar to that of the computed ECD spectrum of (3*S*,4*R*,7*S*)-**5** (Figure 3). Accordingly, the structure of compound **5** was proposed as (+)- (3*S*,4*R*,7*S*)-curvulariahawadride.

Five known compounds (**1**–**4** and **6**) were identified as cochlioquinone N (**1**) [29], cochlioquinone A (**2**) [30], stemphone (**3**) [31], anhydrocochlioquinone A (**4**) [32], and terpestacin (**6**) [33] by comparison of their spectroscopic data with those in the literature. Compounds **1**–**4** were cochlioquinone derivatives containing a tetracyclic quinone skeleton in common. These compounds were isolated from *Curvularia* sp. for the first time. Previous reports showed that these compounds have been isolated from other genera of the *Pleosporaceae* family. Cochlioquinone N (**1**) had been isolated from *Bipolaris* sp. [27], while cochlioquinone A (**2**) had been found from *Bipolaris* and *Cochliobolus* genera, including *B. sorokiniana* [34], *Bipolaris* sp. [27], *B. bicolor* [35], *Cochliobolus* sp. [36], and *C. miyabeanus* [37]. Stemphone (**3**) has been reported from other genera of the *Pleosporaceae* family, including *Drechslera sacchari* [38], *B. bicolor* [35], and *Stemphylium sarcinaeforme* [29]. Anhydrocochlioquinone A (**4**) has also been isolated from *B. oryzae* [30], *B. luttrellii* [39], *Cochliobolus* sp. [36], and *Veronaea* sp. (Incertae sedis) [40]. Terpeatacin (**6**) is a bicyclo sesterterpene containing a 5-membered ring fusing with a 15-membered ring and was isolated for the first time in *Curvularia* sp. Terpeatacin (**6**) had been isolated from plant pathogenic fungi, including *Arthrinium* sp. (Apiosporaceae) [41], *Neofusicoccum batangarum* (Botryosphaeriaceae) [42], *Simplicillium* sp. (Cordycipitaceae) [43], *Embellisia chlamydospora* (Pleosporaceae) [44], *Cochliobolus* sp. (Pleosporaceae) [45], *Fusarium culmorum* (Nectriaceae) [46], and *Bipolaris sorokiniana* (Pleosporaceae) [47].

### 3.3. Cytotoxicity against the Three Cancer Cell Lines, including Lung Cancer A549, Colorectal Cancer SW480, and Leukemic K562 Cells and Mammalian Cells (RAW 264.7 Cells)

Compounds **1**–**6** were also evaluated for their cytotoxicities against the three cancer cell lines, lung cancer A549, colorectal cancer SW480, and leukemic K562 cells, and mammalian cells, RAW 264.7 (Table 2). Compounds **1**, **3**, **5**, and **6** were not cytotoxic at the concentration of 100 µg/mL, and their cell viability was in the range of 71.38 to 99.15%, whereas compounds **2** and **4** were cytotoxic with the cell viability in the range of 8.40 to 14.95%.

Cochlioquinone (**2**) and anhydrocochlioquinone A (**4**) were further evaluated for their cytotoxicities, using the MTT assay at the concentrations of 6.25, 12.5, 25, 50, and 100 μg/mL (Table 3). At all concentrations, cochlioquinone (**2**) and anhydrocochlioquinone A (**4**) showed cytotoxicities against the three cancer cell lines and mammalian cells (RAW 264.7 cells). Cochlioquinone A (**2**) exhibited significant cytotoxicities (Table 3) against lung cancer A549, colorectal cancer SW480, and leukemic K562 cells with IC_50_ values of <11.73, 14.34, <11.73, and 17.59 µM, respectively. Anhydrocochlioquinone A (**4**) also exhibited good cytotoxicities against all cell lines with the same IC_50_ value of <12.14 µM (Table 3). Unfortunately, cochlioquinone (**2**) and anhydrocochlioquinone A (**4**) showed cytotoxicities against mammalian cells, RAW 264.7 cells, in all concentrations with IC_50_ values of <11.73 and <12.14 µM, respectively.

The previous report showed that anhydrocochlioquinone A (**4**) displayed weak cytotoxicity against lung cancer (A549) cell line with the inhibition ratio of 41% at 30 µg/mL [40]. In this study, anhydrocochlioquinone A (**4**) showed the cytotoxicity against lung cancer (A549) cell line with the IC_50_ value of <12.14 µM, which agrees with the previous report. It is interesting to note that compounds **1**-**4** shared the same core structure framework (cochlioquinone derivative); however, the cytotoxicities against the three cancer cell lines were different. Cochlioquinone A (**2**) was different from cochlioquinone N (**1**) (R_11_ = NH_2_) at C-11 and stemphone (**3**) at C-2/3 (∆^2(3)^). The contained amino group at C-11 of cochlioquinone N (**1**) and a double bond at C-2/3 (∆^2(3)^) of stemphone (**3**) have much more effect on reducing the cytotoxicities against the three cancer cell lines. In the case of cochlioquinone A (**2**) and anhydrocochlioquinone A (**4**), cochlioquinone A (**2**) contained a hydroxy group at C-12, while anhydrocochlioquinone A (**4**) was a dehydration form at C-12/13 (∆^12(13)^). This difference had no significant effects on cytotoxicity.

### 3.4. Nitric Oxide Production Inhibitory Activity of Isolated Compounds **1**–**6**

Compounds **1**–**6** were screened for their NO production inhibitory activity. At the concentration of 100 µg/mL, compounds **1**, **3**, **5**, and **6** showed NO production inhibitory with the percentage inhibition ranging from 41.03 to 74.01% and the IC_50_ values ranging from 12.9–53.7 µM (Table 4). Cochlioquinone A (**2**) and anhydrocochlioquinone A (**4**) were not tested due to these compounds leading to cell death. Curvulariahawadride (**5**) showed the best NO production inhibition activity with an IC_50_ value of 12.9 µM, which was better than the positive control, indomethacin (IC_50_ value of 73.4 µM). Cochlioquinone N (**1**) and stemphone (**3**) also showed NO production inhibition activity better than the positive control with IC_50_ values of 53.7 and 32.8, respectively. The other compounds (**2**, **4**, and **6**) were inactive at the concentration of 100 µg/mL. Notably, this is the first publication on the NO production inhibitory activity of these compounds.

## 4. Conclusions

The chemical investigation of plant pathogenic fungus *Curvularia* sp. led to the isolation and identification of a new nonadride derivative, curvulariahawadride (**5**), together with five known compounds. Cochlioquinones were found as major compounds from this study. All compounds were evaluated for their cytotoxicities against the three cancer cell lines, lung cancer A549, colorectal cancer SW480, and leukemic K562 cells, and NO production inhibitory activity. Only two compounds (**2** and **4**) showed cytotoxicities against the three cancer cell lines. In the case of NO production inhibitory activity, compound **5** showed the best NO production inhibitory activity, which better than the positive control.

## Figures and Tables

**Table 1:** ^1^H (400 MHz, CDCl_3_) and ^13^C (100 MHz, CDCl_3_) NMR Spectroscopic data of curvulariahawadride (**5**).

Position	δ_C_	δ_H_ [mult, *J* in Hz]	HMBC (^1^H→^13^C)
1	142.7		
2	146.9		
3	40.4	2.82 (td, 10.5, 2.7)	1, 2, 4, 10, 3′
4	46.3	2.68 (m)	3
5	148.3	6.46 (d, 11.7)	4, 6, 13, 2″
6	131.2		
7	44.9	3.49 (d, 12.0)	5, 6, 8, 9, 12, 13
8	24.3	2.61 (m); 1.76 (dd, 13.0, 12.6)	5, 6, 7, 9, 12
9	24.4	2.93 (dd, 13.0, 7.3); 2.46 (t, 13.0)	1, 2, 7, 8, 11
10	165.9		
11	165.7		
12	177.6		
13	169.8		
1’	14.0	0.92 (t, 7.6)	2’, 3’
2’	21.0	1.25 (m)	3, 1’, 3’
3’	34.9	1.89 (m); 1.53 (m)	1’
1”	12.3	0.93 (t, 7.2)	4, 2”
2”	25.9	1.98 (ddd, 13.2, 7.2, 2.5); 1.38 (m)	4, 5, 1”

**Table 2:** Cell viability of compounds **1**–**6.**

Samples (100 µg/mL)	Cell Viability (%)			
	RAW 264.7	A549 (Lung Cancer)	SW480 (Colorectal Cancer)	K562 (Leukemic Cells)
**1**	97.14 ± 0.81	88.51 ± 1.19	82.60 ± 1.68	81.07 ± 3.80
**2**	10.80 ± 0.09	13.37 ± 0.48	14.95 ± 0.57	12.54 ± 1.24
**3**	71.38 ± 2.67	96.32 ± 3.70	75.59 ± 2.00	79.23 ± 1.86
**4**	11.07 ± 0.43	8.40 ± 0.58	14.39 ± 0.40	10.56 ± 0.20
**5**	82.59 ± 1.72	84.08 ± 3.01	97.87 ± 1.70	75.65 ± 1.19
**6**	99.34 ± 2.51	92.03 ± 3.75	97.69 ± 1.04	99.15 ± 3.84
5% DMSO (control)	100.00 ± 2.47	100.00 ± 0.41	100.00 ± 2.49	100.00 ± 1.89

**Table 3:** Cytotoxicities of Cochlioquinone (**2**) and Anhydrocochlioquinone A (**4**).

Sample	Concentration	Cell Viability (%)			
		RAW 264.7	A549 (Lung Cancer)	SW480 (Colorectal Cancer)	K562 (Leukemic Cells)
**2**	100	10.80 ± 0.09	13.37 ± 0.48	14.95 ± 0.57	12.54 ± 1.24
	50	11.67 ± 0.08	14.77 ± 2.54	15.64 ± 0.77	13.35 ± 0.89
	25	15.00 ± 0.29	17.92 ± 1.93	17.56 ± 0.70	21.01 ± 2.61
	12.5	16.61 ± 0.52	23.07 ± 1.84	23.41 ± 0.25	38.42 ± 3.18
	6.25	28.48 ± 1.98	73.11 ± 1.09	30.75 ± 0.40	70.95 ± 1.69
	IC_50_ (µM)	<11.73	14.34	<11.73	17.59
**4**	100	11.07 ± 0.43	8.40 ± 0.58	14.39 ± 0.40	10.56 ± 0.20
	50	12.79 ± 0.70	9.35 ± 0.52	14.71 ± 0.24	11.27 ± 0.40
	25	12.14 ± 0.42	10.16 ± 0.17	15.37 ± 0.42	12.88 ± 0.34
	12.5	12.45 ± 0.47	11.58 ± 1.05	15.59 ± 0.39	14.03 ± 2.31
	6.25	14.32 ± 0.73	12.39 ± 0.17	17.29 ± 0.33	19.48 ± 2.62
	IC_50_ (µM)	<12.14	<12.14	<12.14	<12.14

**Table 4:** Nitric oxide production inhibition activity of compounds **1**–**6.**

Samples	NO Production Inhibition	
	% of NO Inhibition at 100 µg/mL	IC_50_ (µM)
**1**	51.30 ± 1.65	53.7
**2**	not tested *	not tested *
**3**	73.41 ± 0.44	32.8
**4**	not tested *	not tested *
**5**	74.01 ± 1.14	12.8
**6**	41.03 ± 3.36	inactive
Indomethacin	77.45 ± 0.28	73.4

* Not tested due to compound leads to cell death.

## Data Availability

Data included in article/Supplementary Materials/referenced in the article.

## Supplementary Materials

The following are available online at https://www.mdpi.com/article/10.3390/jof7060408/s1. The ^1^H and ^13^C NMR spectroscopic data of all known compounds. Figure S1: ^1^H NMR (400 MHz, CDCl_3_) of curvulariahawadride (**5**), Figure S2: ^13^C NMR (100 MHz, CDCl_3_) of curvulariahawadride (5), Figure S3: COSY (400 MHz, CDCl_3_) of curvulariahawadride (**5**), Figure S4: NOESY (400 MHz, CDCl_3_) of curvulariahawadride (**5**), Figure S5: HSQC (400 MHz, 100 MHz, CDCl_3_) of curvulariahawadride (**5**), Figure S6: HMBC (400 MHz, 100 MHz, CDCl_3_) of curvulariahawadride (**5**), Figure S7: HRESITOFMS spectrum of curvulariahawadride (**5**), Figure S8: ^1^H NMR (400 MHz, CDCl_3_) of compound 1, Figure S9: ^13^C NMR (100 MHz, CDCl_3_) of compound **1**, Figure S10. ^1^H NMR (400 MHz, acetone-d_6_) of compound **2**, Figure S11: ^13^C NMR (100 MHz, acetone-*d_6_*) of compound **2**, Figure S12: ^1^H NMR (400 MHz, acetone-*d_6_*) of compound 3, Figure S13: ^13^C NMR (100 MHz, acetone-*d_6_*) of compound **3**, Figure S14: ^1^H NMR (400 MHz, CDCl_3_) of compound **4**, Figure S15: ^13^C NMR (100 MHz, CDCl_3_) of compound **4**, Figure S16: ^1^H NMR (400 MHz, CDCl_3_) of compound **6**, Figure S17: ^13^C NMR (100 MHz, CDCl_3_) of compound **6**, Figure S18. Optimized structures of compounds **5**a-**5**f using B3LYP/6-31G(d,p) level in methanol (PCM). Figure S19. ECD spectra of compounds **5**a-**5**f compare with experimental ECD compound **5**.
