# Supplementary material for: Cytotoxicity and Nitric Oxide Production Inhibitory Activities of Compounds Isolated from the Plant Pathogenic Fungus Curvularia sp
Source: J Fungi (Basel). 2021 May 22;7(6):408. doi: 10.3390/jof7060408 (PMC8224630; doi:10.3390/jof7060408)
Supplement: Supplementary file 1 [file jof-07-00408-s001.zip › jof-1234406-supplementary.pdf]

Supplementary Material

Cytotoxicity and nitric oxide production inhibitory activities of compounds isolated from the plant pathogenic fungus *Curvularia* sp.

Virayu Suthiphasilp, Achara Raksat, Tharakorn Maneerat, Sarinya Hadsadee, Siriporn Jungsuttiwong, Stephen G. Pyne, Putarak Chomnunti, Wuttichai Jaidee, Rawiwan Charoensup, Surat Laphookhieo

Content **Page**

The ^{1}H and ^{13}C NMR spectroscopic data of all known compounds S2
Figure S1. ^{1}H NMR (400 MHz, $CDCl_3$) of curvulariahawadride (**5**) S4
Figure S2. ^{13}C NMR (100 MHz, $CDCl_3$) of curvulariahawadride (**5**) S4
Figure S3. COSY (400 MHz, $CDCl_3$) of curvulariahawadride (**5**) S5
Figure S4. NOESY (400 MHz, $CDCl_3$) of curvulariahawadride (**5**) S5
Figure S5. HSQC (400 MHz, 100 MHz, $CDCl_3$) of curvulariahawadride (**5**) S6
Figure S6. HMBC (400 MHz, 100 MHz, $CDCl_3$) of curvulariahawadride (**5**) S6
Figure S7. HRESITOFMS spectrum of curvulariahawadride (**5**) S7
Figure S8. ^{1}H NMR (400 MHz, $CDCl_3$) of compound **1** S7
Figure S9. ^{13}C NMR (100 MHz, $CDCl_3$) of compound **1** S8
Figure S10. ^{1}H NMR (400 MHz, acetone-d_6) of compound **2** S8
Figure S11. ^{13}C NMR (100 MHz, acetone-d_6) of compound **2** S9
Figure S12. ^{1}H NMR (400 MHz, acetone-d_6) of compound **3** S9
Figure S13. ^{13}C NMR (100 MHz, acetone-d_6) of compound **3** S10
Figure S14. ^{1}H NMR (400 MHz, $CDCl_3$) of compound **4** S10
Figure S15. ^{13}C NMR (100 MHz, $CDCl_3$) of compound **4** S11
Figure S16. ^{1}H NMR (400 MHz, $CDCl_3$) of compound **6** S11
Figure S17. ^{13}C NMR (100 MHz, $CDCl_3$) of compound **6** S12
Figure S18. Optimized structures of compounds **5a**-**5f** using B3LYP/6-31G(d,p) level in methanol (PCM) S13
Figure S19. ECD spectra of compounds **5a**-**5f** compare with experimental ECD compound **5** S14

The ^{1}H and ^{13}C NMR spectroscopic data of all known compounds are summarized below.

Cochlioquinone N (**1**): Red needles. mp 185-187 °C; ^{1}H NMR ($CDCl_3$, 400 MHz) δ_H 5.06 (1H, m, H-4), 4.91 (1H, d, *J* = 10.5 Hz, H-12), 3.76 (1H, s, OH-12), 3.25 (1H, dd, *J* = 11.7, 2.6 Hz, H-21), 3.14 (1H, dd, *J* =11.7, 3.7 Hz, H-17), 2.50 (1H, m, H-19), 2.48 (1H, m, H-5), 2.14 (1H, s, H-30), 2.10 (1H, m, H-15), 1.94 (1H, m, H-15), 1.78 (1H, m, H-16), 1.69 (1H, d, *J* = 10.5 Hz, H-13), 1.66 (1H, m, H-20), 1.54 (1H, m, H-16), 1.42 (2H, m, H-3, H-20), 1.40 (2H, m, H-2, H-19), 1.33 (3H, s, H-26), 1.19 (3H, s, H-24), 1.17 (3H, s, H-23), 1.06 (1H, m, H-2), 1.02 (3H, s, H-25), 1.02 (3H, d, *J* = 7.3 Hz, H-27), 0.84 (3H, t, *J* = 7.2 Hz, H-1), and 0.78 (3H, d, *J* = 6.6 Hz, H-28); ^{13}C NMR ($CDCl_3$, 100 MHz) δ_C 183.9 (C-7), 178.7 (C-10), 169.4 (C-29), 154.2 (C-8), 144.8 (C-6), 110.9 (C-11), 85.0 (C-21), 83.7 (C-14), 83.5 (C-17), 80.6 (C-4), 71.8 (C-22), 63.1 (C-12), 51.7 (C-13), 38.6 (C-5, C-19), 37.4 (C-15), 36.7 (C-3, C-18), 26.0 (C-24), 25.1 (C-2, C-16), 23.7 (C-23), 21.5 (C-20), 21.2 (C-26), 21.0 (C-30), 15.1 (C-28), 13.7 (C-27), 12.6 (C-25), 10.9 (C-1).

Cochlioquinone A (**2**): Colorless solid. m.p. 130-132 °C; ^{1}H NMR (acetone-d_6, 400 MHz) δ_H 6.62 (1H, s, H-11), 4.93 (2H, m, H-4, H-12), 4.08 (1H, m, OH-12), 3.36 (1H, dd, *J* = 7.7, 6.1 Hz, H-5), 3.21 (2H, m, H-17, H-21), 2.48 (1H, m, H-19), 2.00 (3H, s, H-30), 1.99 (1H, m, H-15), 1.91 (1H, m, H-15), 1.69 (1H, m H-13), 1.66 (1H, m, H-20), 1.63 (1H, m H-3), 1.55 (1H, m, H-16), 1.49 (1H, m, H-20), 1.43 (1H, m, H-19), 1.32 (3H, s, H-26), 1.14 (3H, s, H-23), 1.13 (3H, s, H-27, H-24), 1.08 (1H, m, H-16), 1.04 (3H, s, H-25), and 0.87 (6H, m, H-1, H-28); ^{13}C NMR (acetone-d_6, 100 MHz) δ_C 188.1 (C-10), 181.4 (C-7), 169.9 (C-29), 151.2 (C-8), 147.8 (C-6), 133.9 (C-11), 119.7 (C-9), 85.4 (C-21), 83.7 (C-17), 82.6 (C-14), 79.4 (C-4), 70.8 (C-22), 62.6 (C-12), 52.1 (C-13), 38.6 (C-19), 37.4 (C-15), 36.5 (C-18), 36.1 (C-3), 32.9 (C-5), 25.1 (C-2), 24.9 (C-24), 24.8 (C-23), 23.3 (C-16), 21.2 (C-20), 20.4 (C-26), 19.9 (C-30), 17.1 (C-27), 15.1 (C-28), 12.0 (C-25), and 10.6 (C-1).

Stemphone (**3**): Yellow solid, m.p. 159-161 °C; ^{1}H NMR (acetone-d_6, 400 MHz) δ_H 6.54 (1H, s, H-11), 5.59 (1H, m, H-2), 5.21 (1H, d, *J* = 9.1 Hz, H-4), 4.93 (2H, dt, *J* = 10.5, 2.4 Hz, H-12), 4.02 (1H, d, *J* = 2.4 Hz, OH-12), 3.33 (1H, m, H-5), 3.24 (1H, m, H-21), 3.21 (1H, m, H-17), 2.47 (1H, m, H-19), 1.99 (1H, m, H-15), 1.89 (3H, s, H-30), 1.88 (1H, m, H-15), 1.73 (1H, m, H-16), 1.71 (1H, m, H-13), 1.66 (1H, m, H-20), 1.63 (3H, d, *J* = 7.4 Hz, H-1), 1.60 (4H, m, H-16, H-28), 1.49 (1H, m, H-20), 1.43 (1H, m, H-19), 1.32 (3H, m, H-26), 1.13 (3H, s, H-23), 1.12 (3H, s, H-24), 1.07 (3H, s, H-25), and 1.03 (3H, d, *J* = 6.7 Hz, H-27); ^{13}C NMR (acetone-d_6, 100 MHz) δ_C 188.0 (C-10), 181.3 (C-7), 169.0 (C-29), 151.2 (C-8), 148.2 (C-6), 132.8 (C-11), 132.5 (C-3), 124.6 (C-2), 119.7 (C-9), 85.3 (C-21), 83.7 (C-17), 82.6 (C-14), 81.2 (C-4), 70.8 (C-22), 62.6 (C-12), 52.2 (C-13), 38.6 (C-19), 37.4 (C-15), 36.5 (C-18), 25.1 (C-16), 24.9 (C-24), 24.8 (C-23), 21.2 (C-20), 20.4 (C-26), 19.9 (C-30), 16.0 (C-25), 12.2 (C-28), 12.0 (C-27), and 10.7 (C-1).

Anhydrocochlioquinone A (**4**): Red amorphous solid. ^{1}H NMR ($CDCl_3$, 400 MHz) δ_H 6.63 (1H, s, H-11), 6.32 (1H, s, H-12), 4.93 (1H, dd, *J* = 7.5, 5.4 Hz, H-4), 3.40 (1H, qd, *J* = 7.5, 5.4 Hz, H-5), 3.19 (2H, ddd, *J* = 11.8, 4.8, 3.2 Hz, H-17, H-21), 2.31 (1H, dt, *J* = 13.3, 3.2 Hz, H-15), 2.11 (1H, m, H-19), 2.04 (4H, m, H-15, H-30), 1.82 (1H, m, H-16), 1.71 (2H, m, H-16, H-20), 1.59 (1H, m, H-20), 1.56 (3H, m, H-26), 1.55 (1H, m, H-19), 1.51 (1H, m, H-3), 1.43 (1H, m, H-2), 1.20 (3H, s, H-23), 1.18 (3H, s, H-24), 1.12 (3H, s, H-25), 1.11 (3H, m, H-27), 1.07

(1H, m, H-2), 0.85 (6H, m, H-1, H-28); ^{13}C NMR ($CDCl_3$, 100 MHz) δ_C 184.9 (C-10), 181.1 (C-7), 170.6 (C-29), 148.9 (C-8), 148.3 (C-6), 147.5 (C-13), 132.9 (C-11), 117.2 (C-9), 110.5 (C-12), 84.6 (C-21), 81.5 (C-14), 81.1 (C-17), 80.0 (C-4), 71.8 (C-22), 38.6 (C-18), 37.8 (C-15), 36.4 (C-3), 34.8 (C-19), 32.5 (C-5), 27.1 (C-26), 26.1 (C-23), 24.5 (C-16), 23.9 (C-24), 23.8 (C-2), 21.7 (C-20), 20.9 (C-30), 20.2 (C-25), 18.3 (C-27), 15.4 (C-28), and 11.1 (C-1).

Terpestacin (**6**): Colorless solid. m.p. 171-172 °C; ^1H NMR ($CDCl_3$, 400 MHz) δ_H 5.40 (1H, m, H-13), 5.24 (1H, m, H-7), 5.12 (1H, m, H-3), 4.06 (1H, dd, *J* = 9.5, 3.5 Hz, H-11), 3.88 (1H, dd, *J* = 10.3, 7.0 Hz, H-24), 3.81 (1H, dd, *J* = 10.4, 5.4 Hz, H-24), 2.72 (1H, m, H-15), 2.65 (1H, m, H-23), 2.44 (1H, m, H-14), 2.38 (1H, m, H-2), 2.26 (1H, m, H-5), 2.25 (1H, m, H-6), 2.11 (1H, m, H-6), 2.09 (1H, m, H-9), 2.00 (1H, m, H-5), 1.90 (1H, m, H-14), 1.78 (1H, m, H-9), 1.74 (2H, m, H-10), 1.70 (1H, m, H-2), 1.64 (3H, s, H-21), 1.63 (3H, s, H-20), 1.57 (3H, s, H-22), 1.30 (3H, d, *J* = 7.1 Hz, H-25), and 1.00 (3H, s, H-19); ^{13}C NMR ($CDCl_3$, 100 MHz) δ_C 208.0 (C-18), 149.1 (C-16), 146.7 (C-17), 138.0 (C-12), 136.4 (C-4), 132.9 (C-8), 128.9 (C-13), 124.3 (C-3), 121.6 (C-7), 76.5 (C-11), 66.1 (C-24), 49.6 (C-15), 48.9 (C-1), 40.3 (C-5), 39.3 (C-2), 37.1 (C-23), 34.9 (C-9), 29.8 (C-10), 28.8 (C-14), 23.8 (C-6), 16.2 (C-19), 15.6 (C-21), 15.3 (C-20), 14.4 (C-25), and 10.4 (C-22).

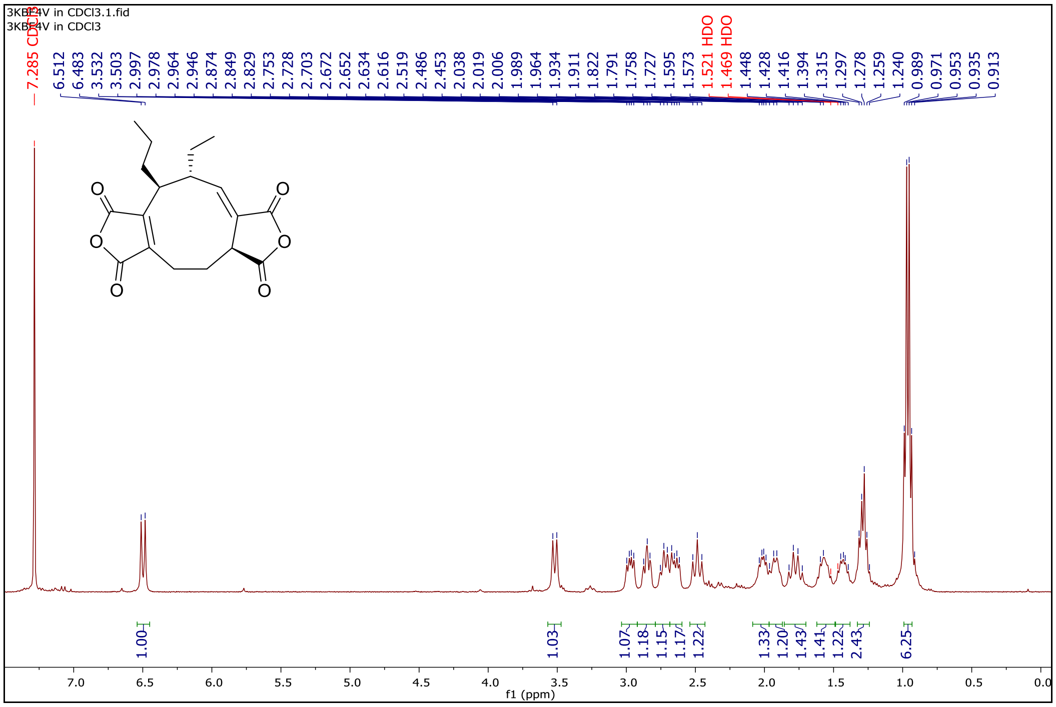

Figure S1. ^{1}H NMR (400 MHz, $CDCl_3$) of curvulariahawadride (**5**)

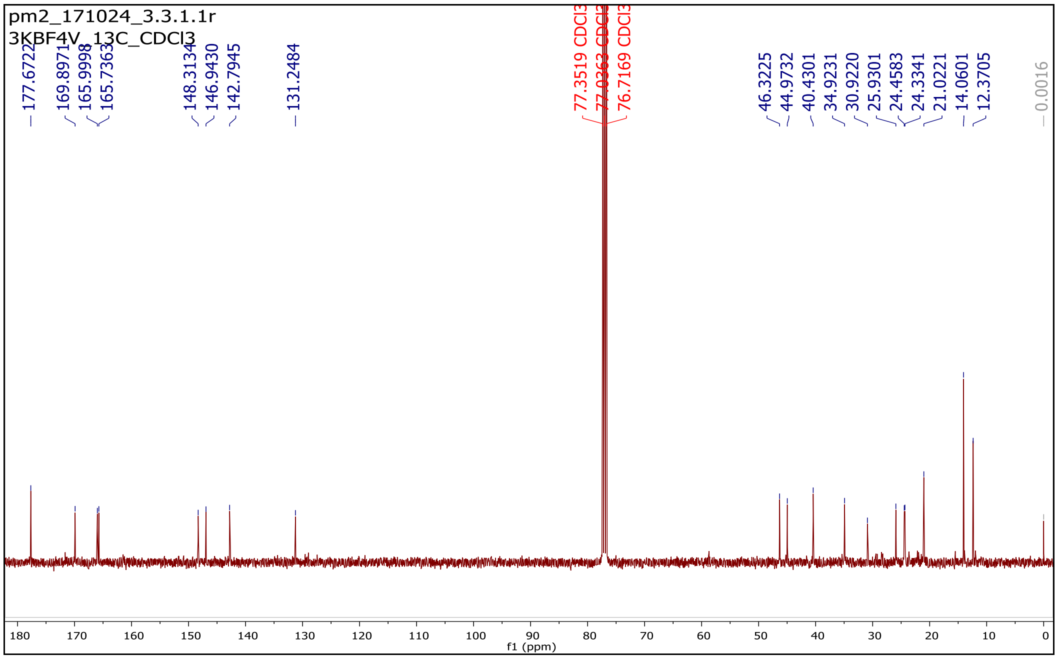

Figure S2. ^{13}C NMR (100 MHz, $CDCl_3$) of curvulariahawadride (**5**)

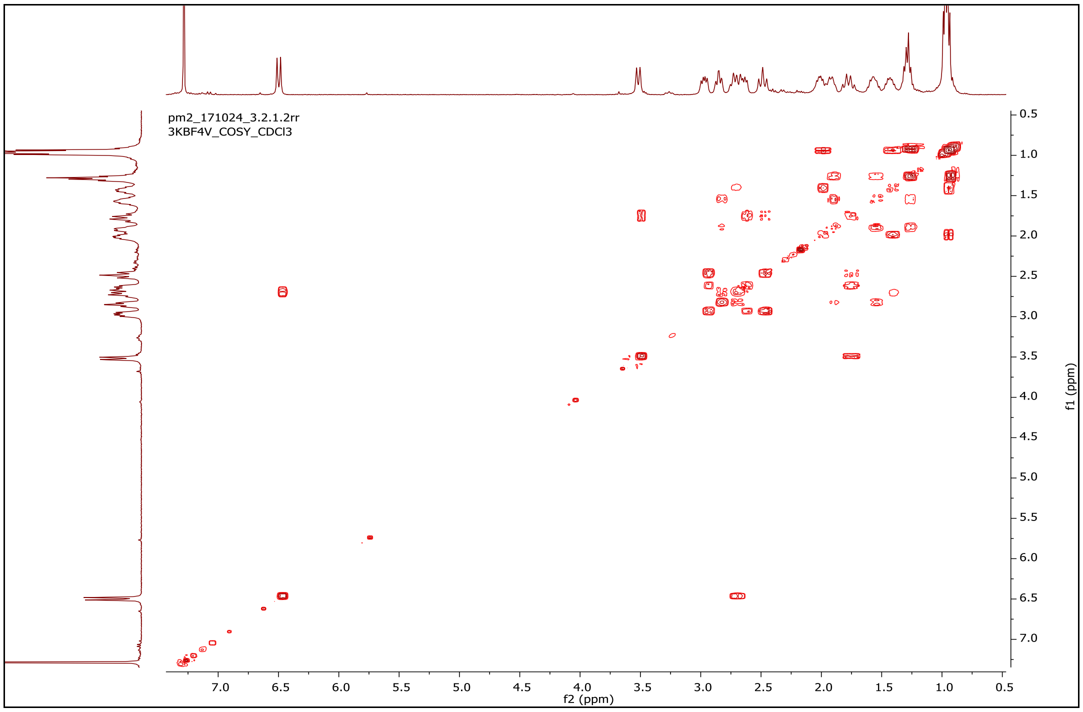

Figure S3. COSY (400 MHz, $CDCl_3$) of curvulariahawadride (**5**)

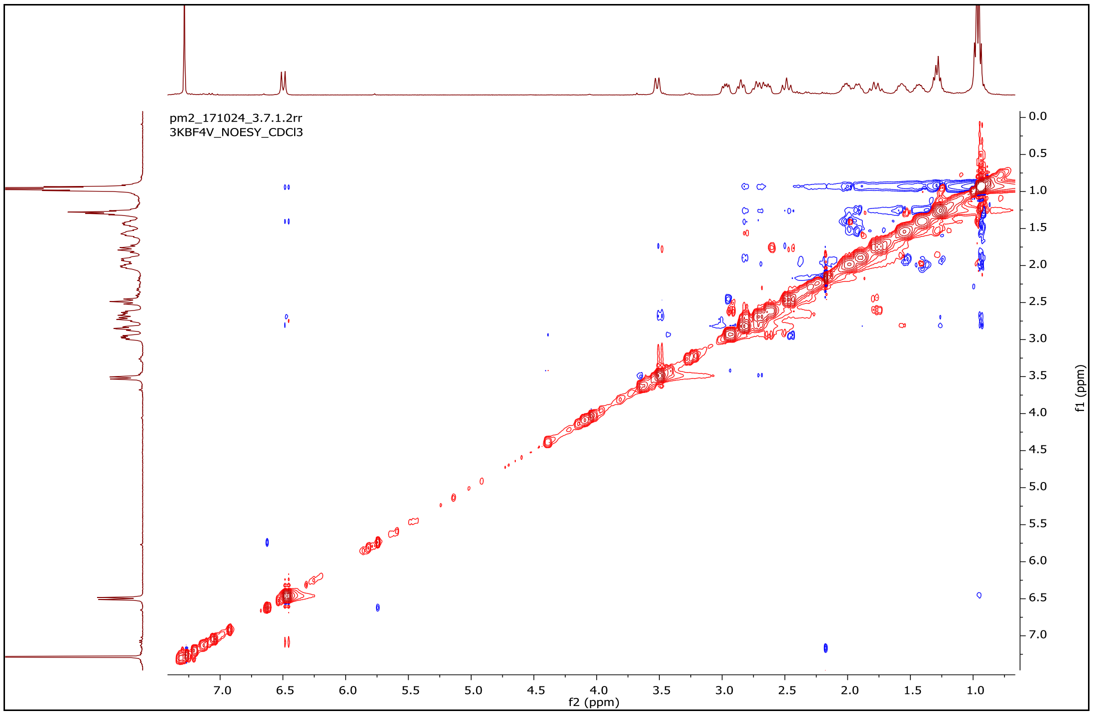

Figure S4. NOESY (400 MHz, $CDCl_3$) of curvulariahawadride (**5**)

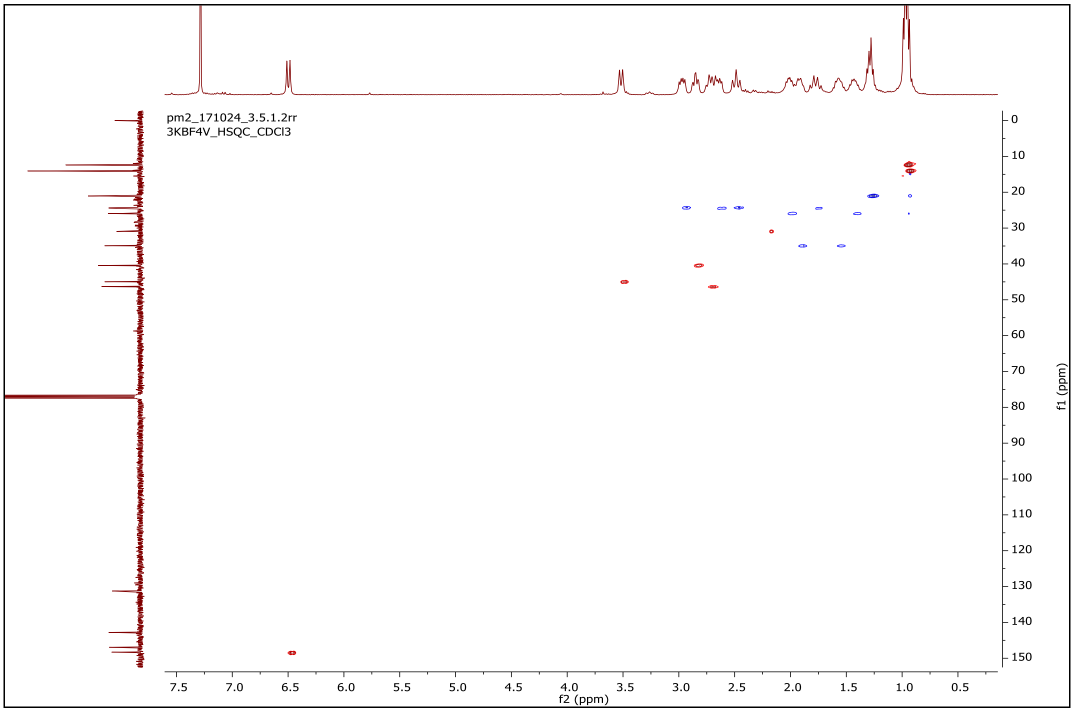

Figure S5. HSQC (400 MHz, 100 MHz, $CDCl_3$) of curvulariahawadride (**5**)

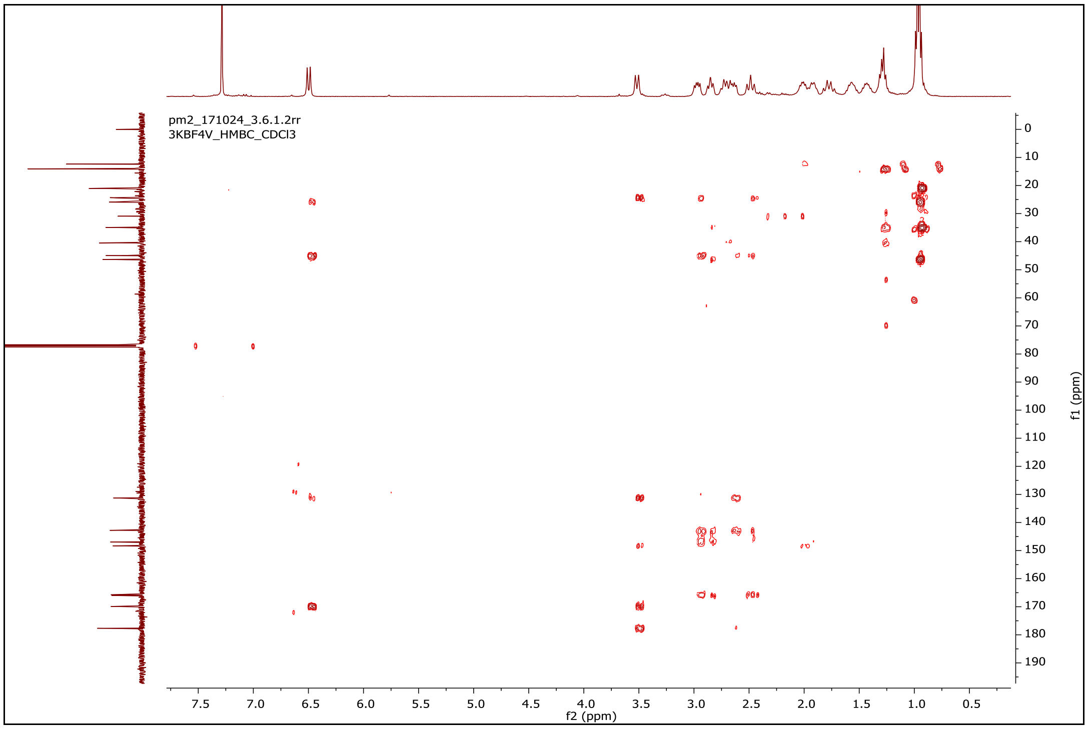

Figure S6. HMBC (400 MHz, 100 MHz, $CDCl_3$) of curvulariahawadride (**5**)

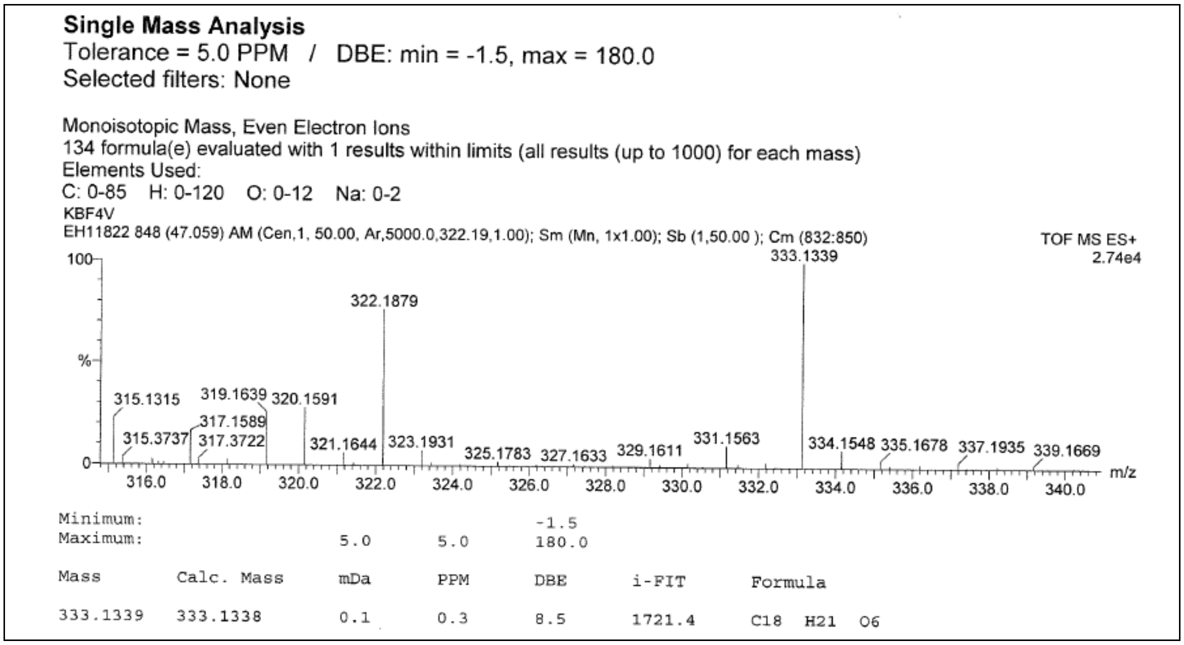

Figure S7. HRESITOFMS spectrum of curvulariahawadride (**5**)

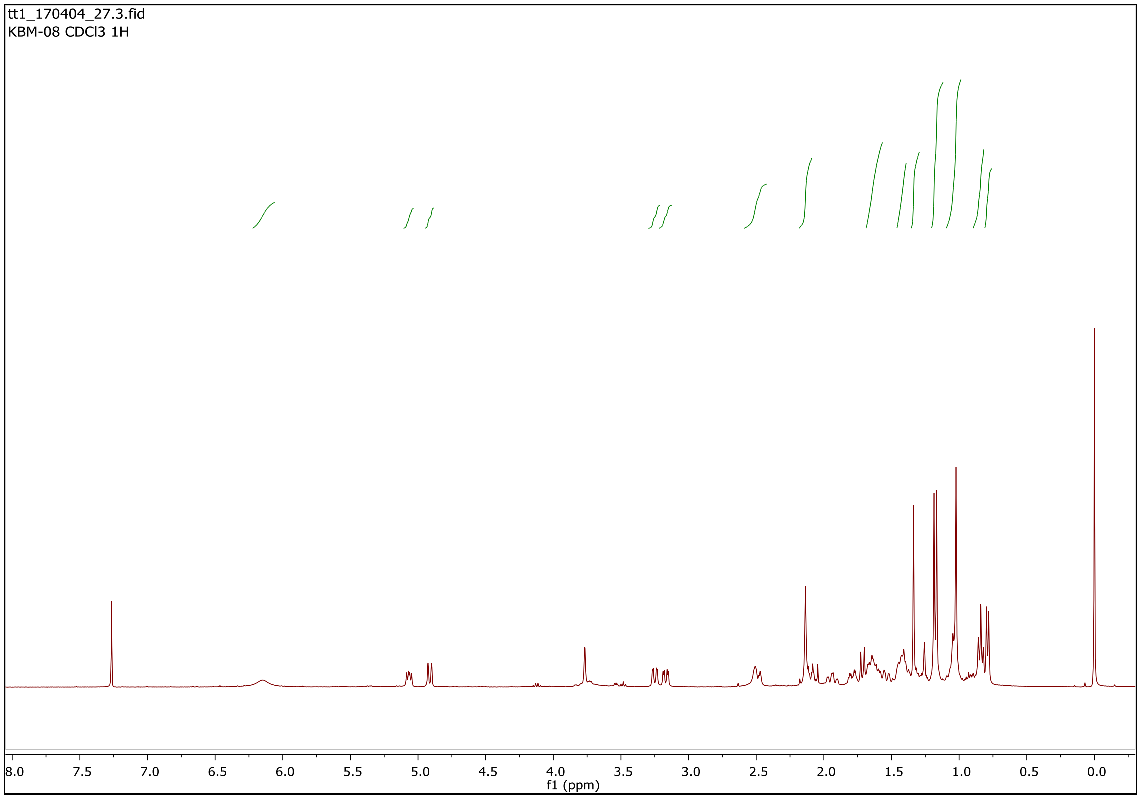

Figure S8. ^{1}H NMR (400 MHz, $CDCl_3$) of compound **1**

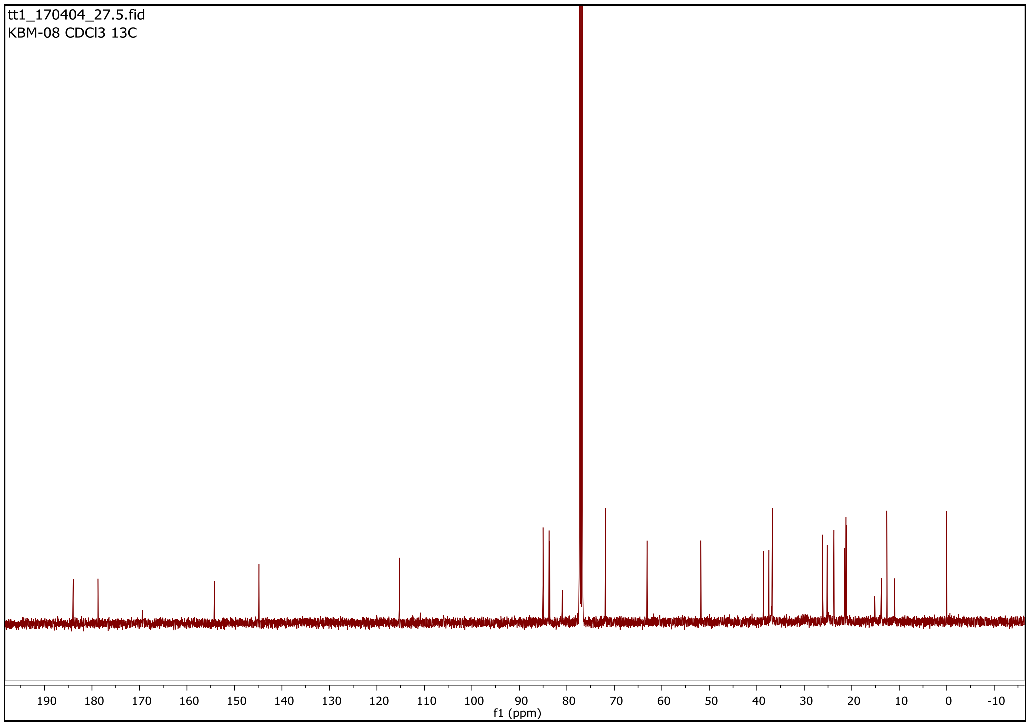

Figure S9. ^{13}C NMR (100 MHz, $CDCl_3$) of compound **1**

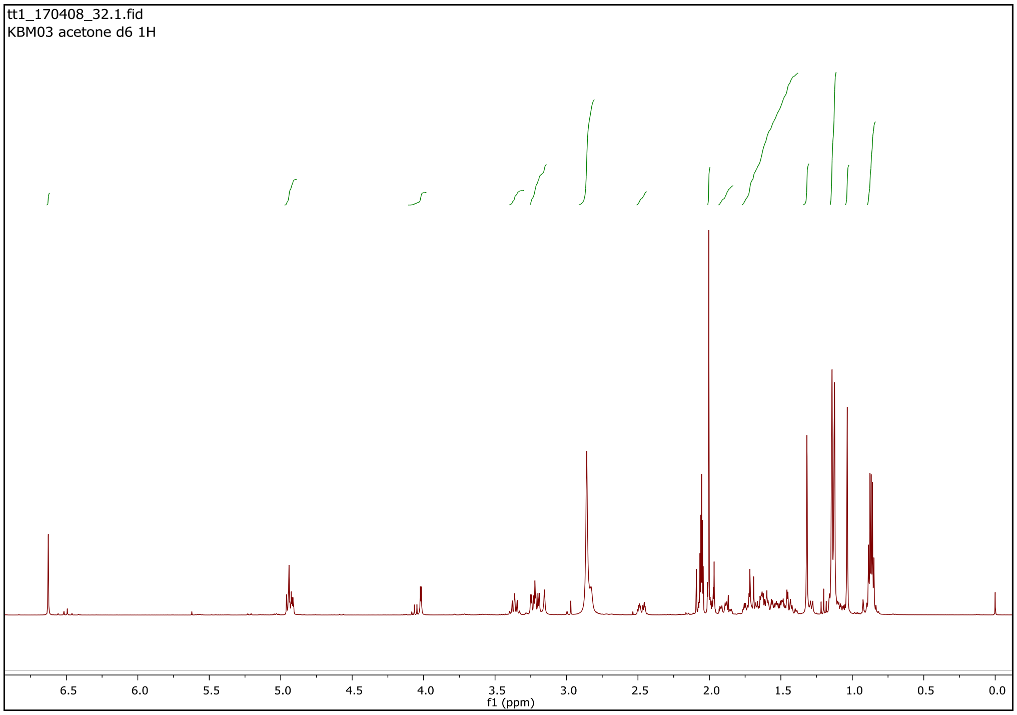

Figure S10. ^{1}H NMR (400 MHz, acetone-d_6) of compound **2**

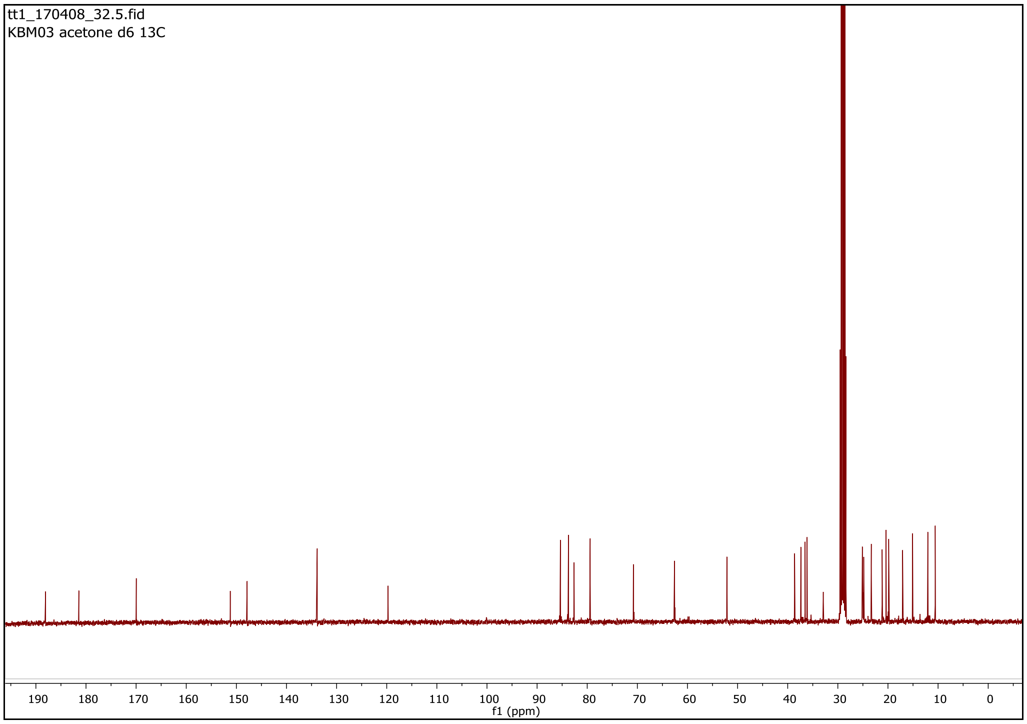

Figure S11. ^{13}C NMR (100 MHz, acetone-d_6) of compound **2**

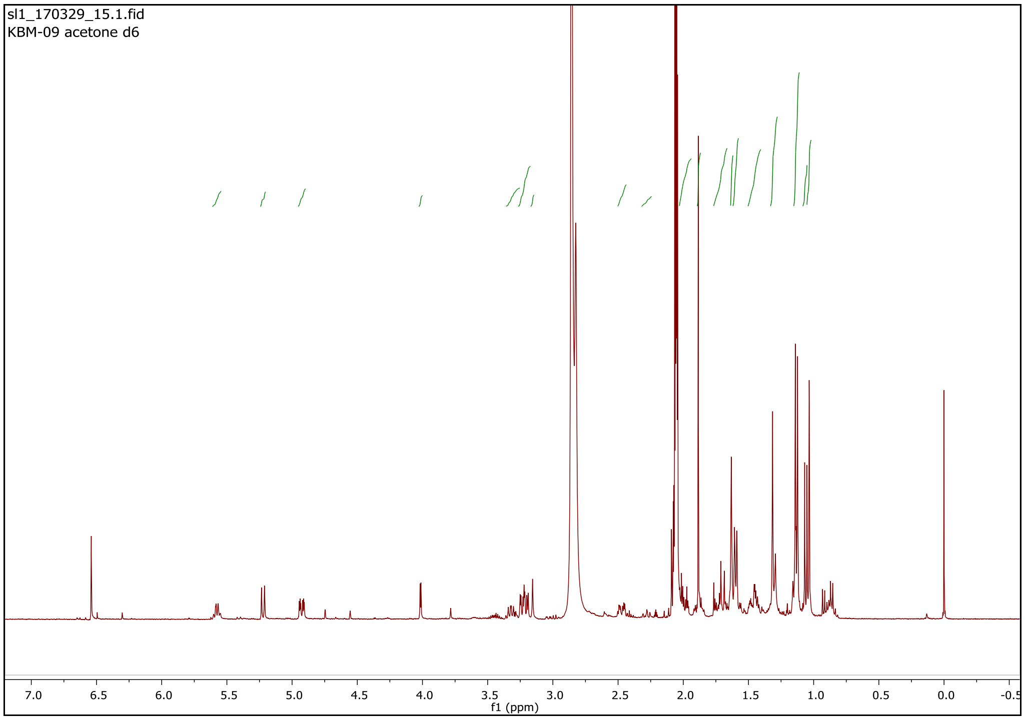

Figure S12. ^{1}H NMR (400 MHz, acetone-d_6) of compound **3**

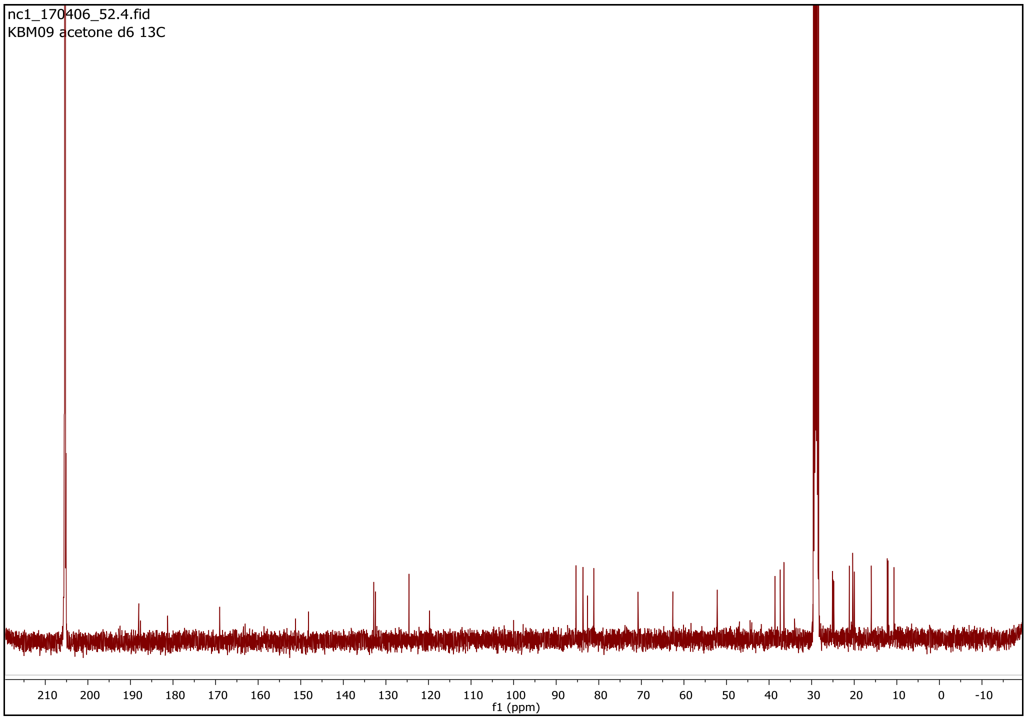

Figure S13. ^{13}C NMR (100 MHz, acetone-d_6) of compound **3**

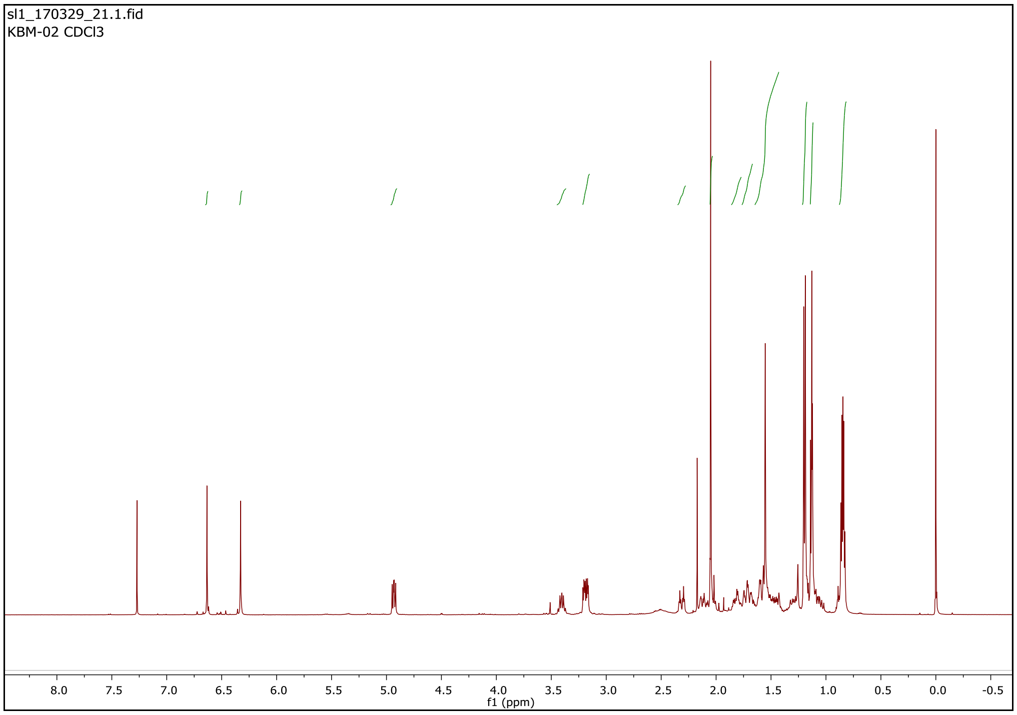

Figure S14. ^{1}H NMR (400 MHz, $CDCl_3$) of compound **4**

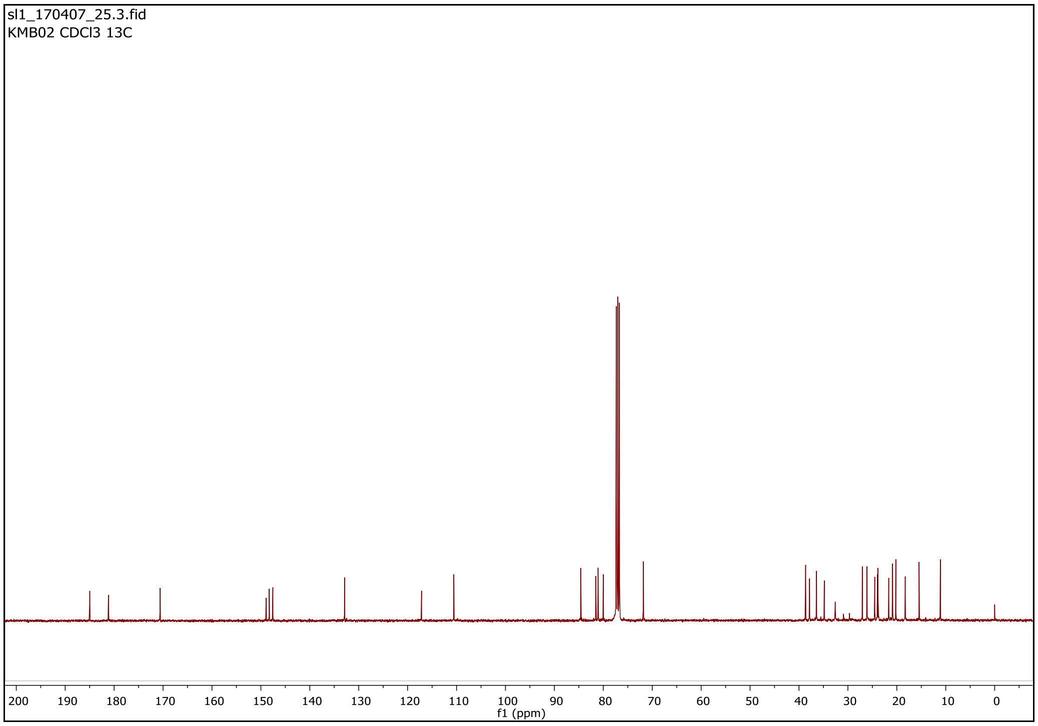

Figure S15. ^{13}C NMR (100 MHz, $CDCl_3$) of compound **4**

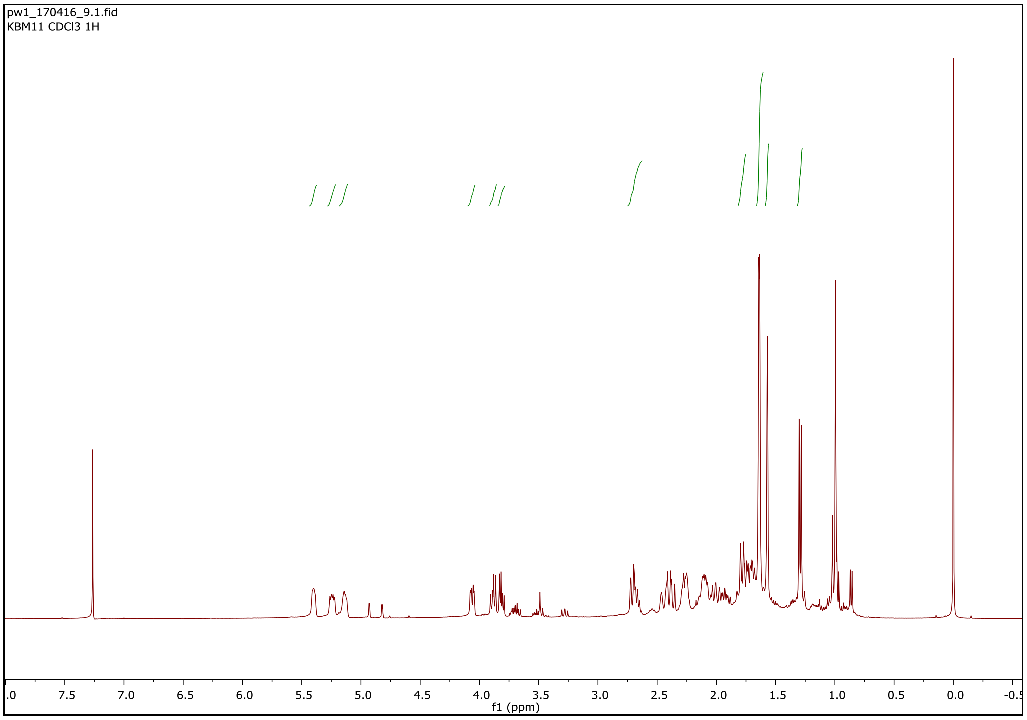

Figure S16. ^{1}H NMR (400 MHz, $CDCl_3$) of compound **6**

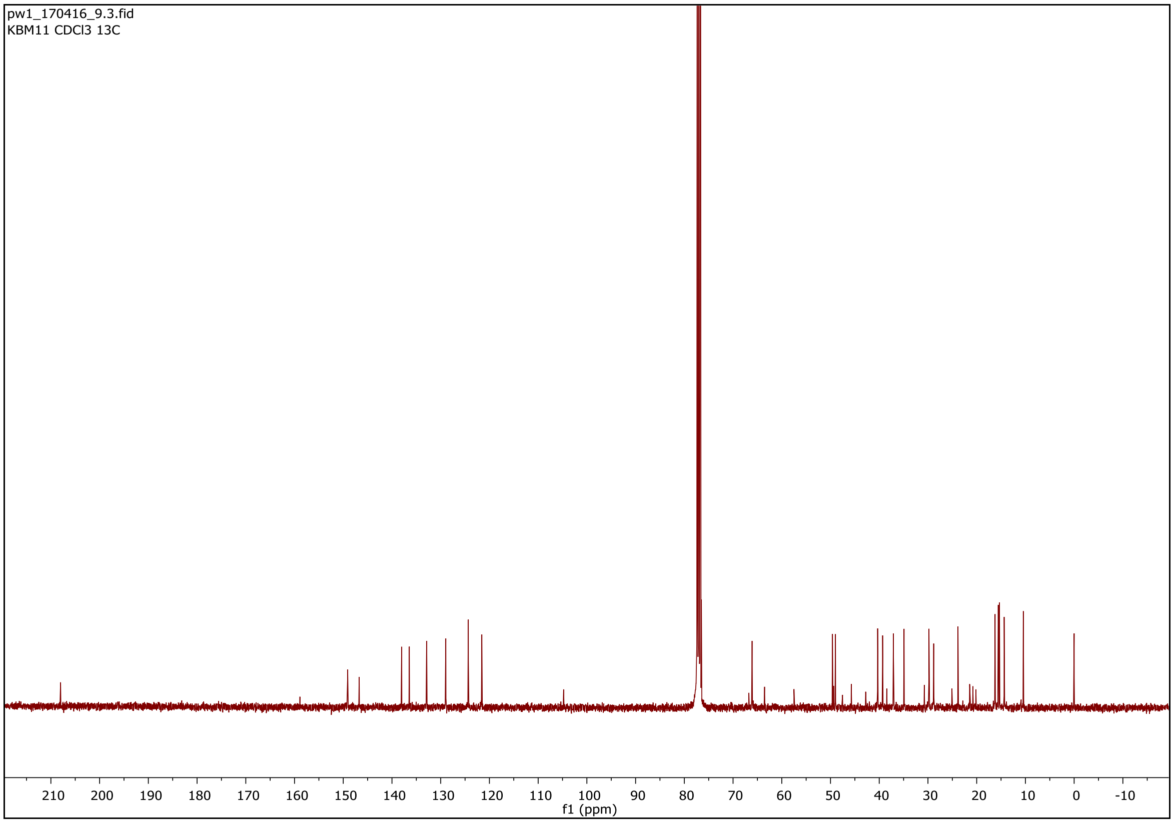

Figure S17. ^{13}C NMR (100 MHz, $CDCl_3$) of compound **6**

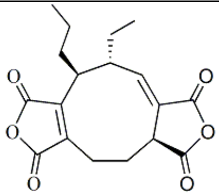 **5a** 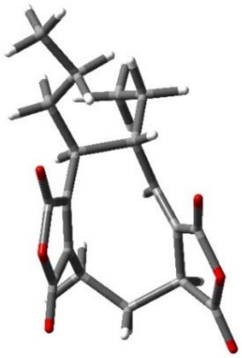	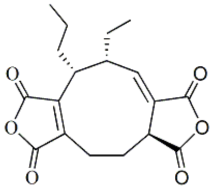 **5b** 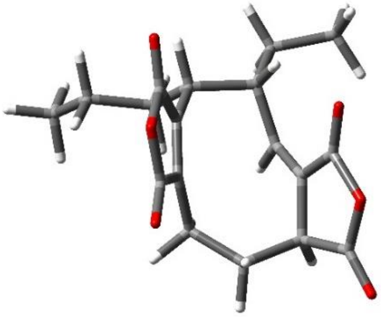	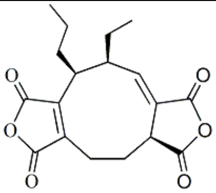 **5c** 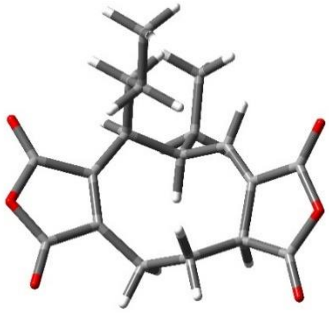
ΔE= 7.16 kcal/mol	ΔE= 0.39 kcal/mol	ΔE= 1.95 kcal/mol
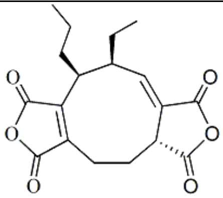 **5d** 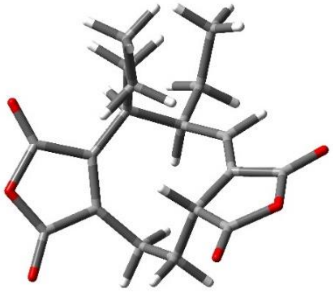	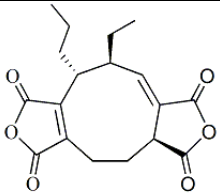 **5e** 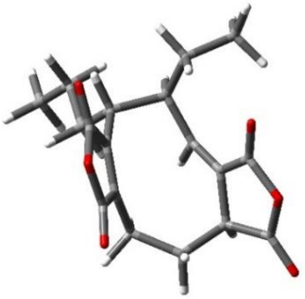	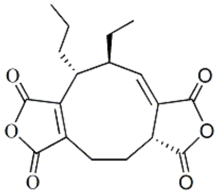 **5f** 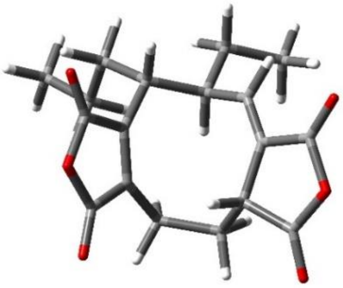
ΔE= 4.24 kcal/mol	ΔE= 5.40 kcal/mol	ΔE= 0.00 kcal/mol

Figure S18. Optimized structures of compounds **5a-5f** using B3LYP/6-31G(d,p) level in methanol (PCM).

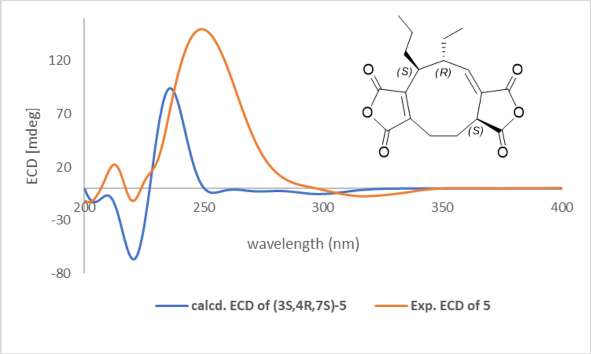

Fig. 19a. Experimental and TDDFT calculated CD spectra of compound **5a** at CAM-B3LYP/ 6-311+ + G(d,p) level in methanol (PCM).

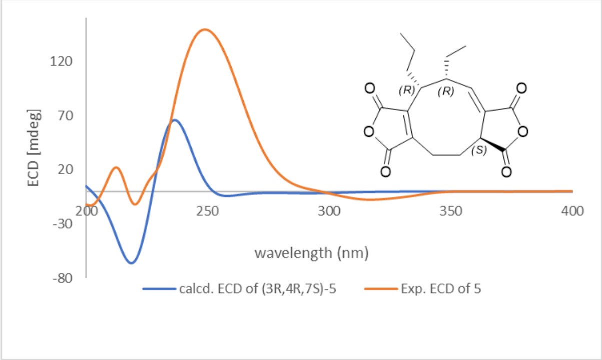

Fig. 19b. Experimental and TDDFT calculated CD spectra of compound **5b** at CAM-B3LYP/6-311++G(d,p) level in methanol (PCM).

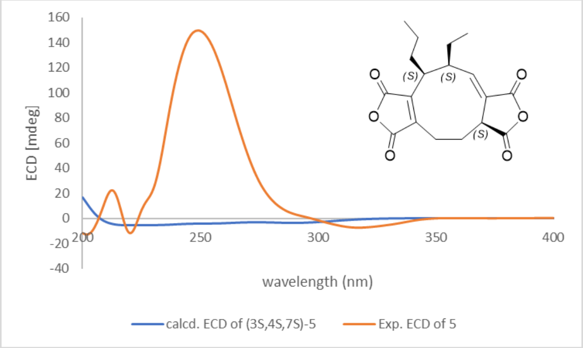

Fig. 19c. Experimental and TDDFT calculated CD spectra of compound **5c** at CAM-B3LYP/ 6-311++G(d,p) level in methanol (PCM).

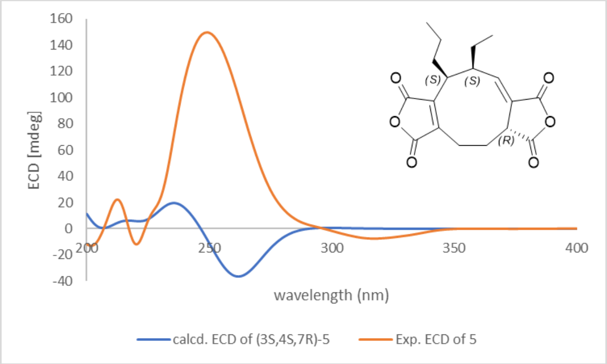

Fig. 19d. Experimental and TDDFT calculated CD spectra of compound **5d** at CAM-B3LYP/6-311++G(d,p) level in methanol (PCM).

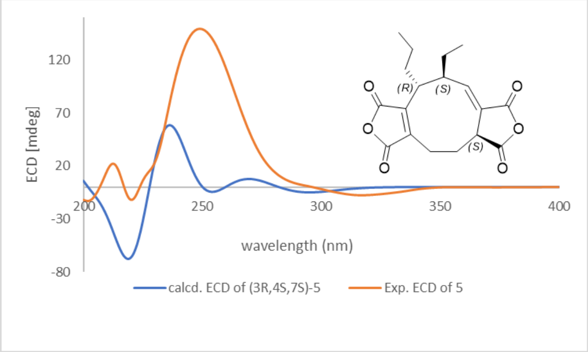

Fig. 19e. Experimental and TDDFT calculated CD spectra of compound **5e** at CAM-B3LYP/ 6-311++G(d,p) level in methanol (PCM).

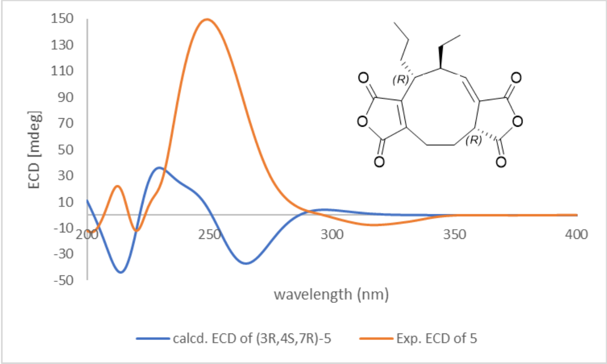

Fig. 19f. Experimental and TDDFT calculated CD spectra of compound **5f** at CAM-B3LYP/6-311++G(d,p) level in methanol (PCM).

Figure S19. ECD spectra of compounds **5a-5f** compare with experimental ECD compound **5**.
